# Gastric carcinoma of the fundic gland type developed 32 years after *Helicobacter pylori* eradication for duodenal ulcer: a case report

**DOI:** 10.1097/MS9.0000000000002192

**Published:** 2024-05-20

**Authors:** Daisuke Suto, Kiichi Sato, Masashi Yoshida, Hiroyoshi Ota, Takaaki Otake, Yosuke Osawa, Yoshimasa Nakazato, Hironori Ohdaira, Yutaka Suzuki, Yutaka Kohgo

**Affiliations:** a Department of Gastroenterology; b Department of Surgery; c Department of Pathology, International University of Health and Welfare Hospital, Nasushiobara, Tochigi; d Department of Biomedical Laboratory Sciences, Shinshu University School of Medicine, Matsumoto, Japan

**Keywords:** case report, duodenal ulcer, endoscopic submucosal dissection, eradication of Helicobacter pylori, esophagogastroduodenoscopy, gastric carcinoma of the fundic gland type

## Abstract

**Introduction:**

Gastric cancer has been reported to occur with mild to moderate mucosal atrophy, particularly after the eradication of *Helicobacter pylori* (*HP*) more than 10 years previously. However, no conclusion has been reached on how many years of esophagogastroduodenoscopy should be performed after *HP* eradication.

**Presentation of case:**

This was a case of gastric carcinoma of the fundic gland type (GCFGT) 32 years after the eradication of *HP*, which is the longest posteradication period reported. A 62-year-old male patient was diagnosed with GCFGT after *HP* eradication and regular esophagogastroduodenoscopy, which revealed a white raised lesion on the anterior wall of the upper part of the body. Endoscopic submucosal dissection was performed for GCFGT, and the vertical and horizontal margins were negative.

**Clinical discussion:**

In this case, *HP* was eradicated in 1990, and GCFGT developed 32 years later. To the best of our knowledge, this is the longest case in which gastric cancer appeared after *HP* eradication. *HP* eradication therapy for a duodenal ulcer was first reported in 1990, supporting that this is the longest case.

**Conclusions:**

This is the first case of gastric cancer more than 20 years after the eradication of *HP*. The endoscopic findings of this case are typical of GCFGT and may be useful when encountering such cases in the future. Therefore, the risk of gastric cancer should be considered for an extended period even after the eradication of *HP*, and regular esophagogastroduodenoscopy is recommended even after the eradication of *HP*.

## Introduction

Highlights

- The patient developed gastric carcinoma of fundic gland type 32 years after eradication of *Helicobacter pylori* for a duodenal ulcer.
- Gastric carcinoma of fundic gland type is a differential diagnosis for white elevated lesions of the stomach.
- The possibility of gastric cancer should be considered for long time after *HP* eradication.

*Helicobacter pylori* (*HP*) infection is a major carcinogenic factor of gastric cancer^1^. *HP* eradication therapy reduces the risk of gastric cancer occurring in relation to atrophic gastritis^2^. Additionally, *HP* eradication therapy for chronic gastritis was approved for coverage by the Japanese national health insurance scheme in February 2013^3^. Although a 30–40% reduction in the risk of gastric cancer has been reported^4^, gastric cancer is reported to occur at an annual rate of 0.2% even after *HP* eradication^5^. Kamada *et al*.^6^ reported that gastric cancer can still occur more than 10 years after *HP* eradication.

No conclusion has been reached on the duration for which esophagogastroduodenoscopy should be performed after *HP* eradication, although the incidence of gastric cancer in patients with a history of duodenal ulcers is low^7^. The occurrence of gastric carcinoma of the fundic gland type (GCFGT) has been reported after *HP* eradication^8^.

Considering the increase in *HP* eradication, gastric cancer after *HP* eradication is expected to increase in the future. We report a case of GCFGT discovered 32 years after *HP* eradication for a duodenal ulcer that was treated using endoscopic submucosal dissection (ESD).

## Presentation of case

This case was approved by the Ethics Committee of the International University of Health and Welfare Hospital (approval number: 23-B-21) and adhered to the principles outlined in the Declaration of Helsinki. Written informed consent was obtained from the patient for the publication of this case report and any accompanying images. This case report has been described in accordance with Surgical CAse REport (SCARE) Criteria^9^.

A male patient in his early 60s underwent regular esophagogastroduodenoscopy after the eradication of *HP*. He underwent *HP* eradication therapy 32 years previously for a duodenal ulcer. The patient has a history of hyperuricemia and hyperlipidemia. His lifestyle includes consuming two shots of alcohol daily and not smoking cigarettes, and he has no family history of any special significance.

**Figure 1:**
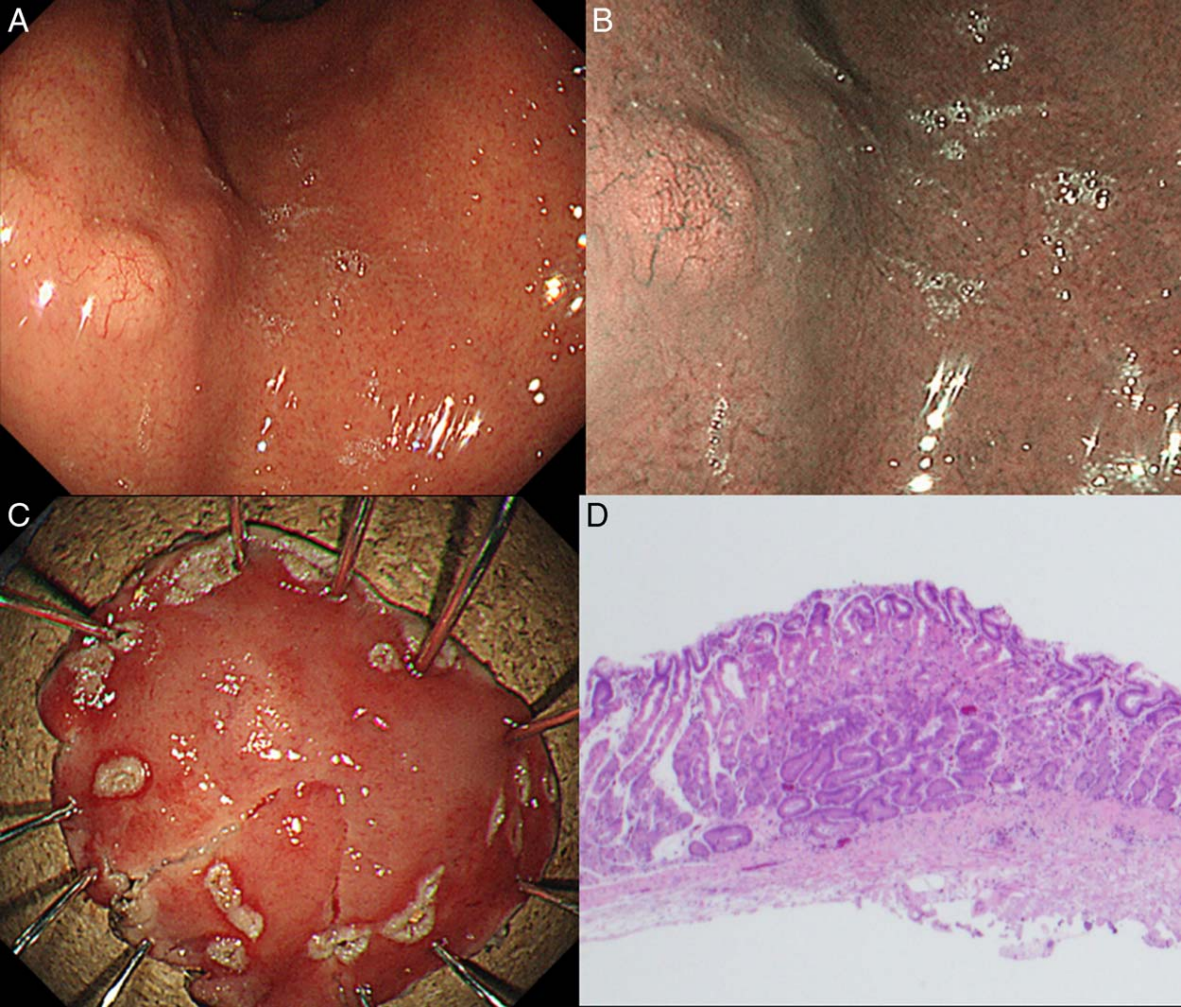
Endoscopic images of gastric carcinoma of the fundic gland type and hematoxylin and eosin (H&E) stained sections. (A) The lesion presents as a submucosal tumor-like white elevated lesion 5 mm in size. (B) Narrow band imaging observation shows dendritic vessels. (C) Endoscopic resection specimen. (D) Pathological examination of the resection specimen showed no vascular and lymphatic invasion (H&E staining, 4×).

**Figure 2:**
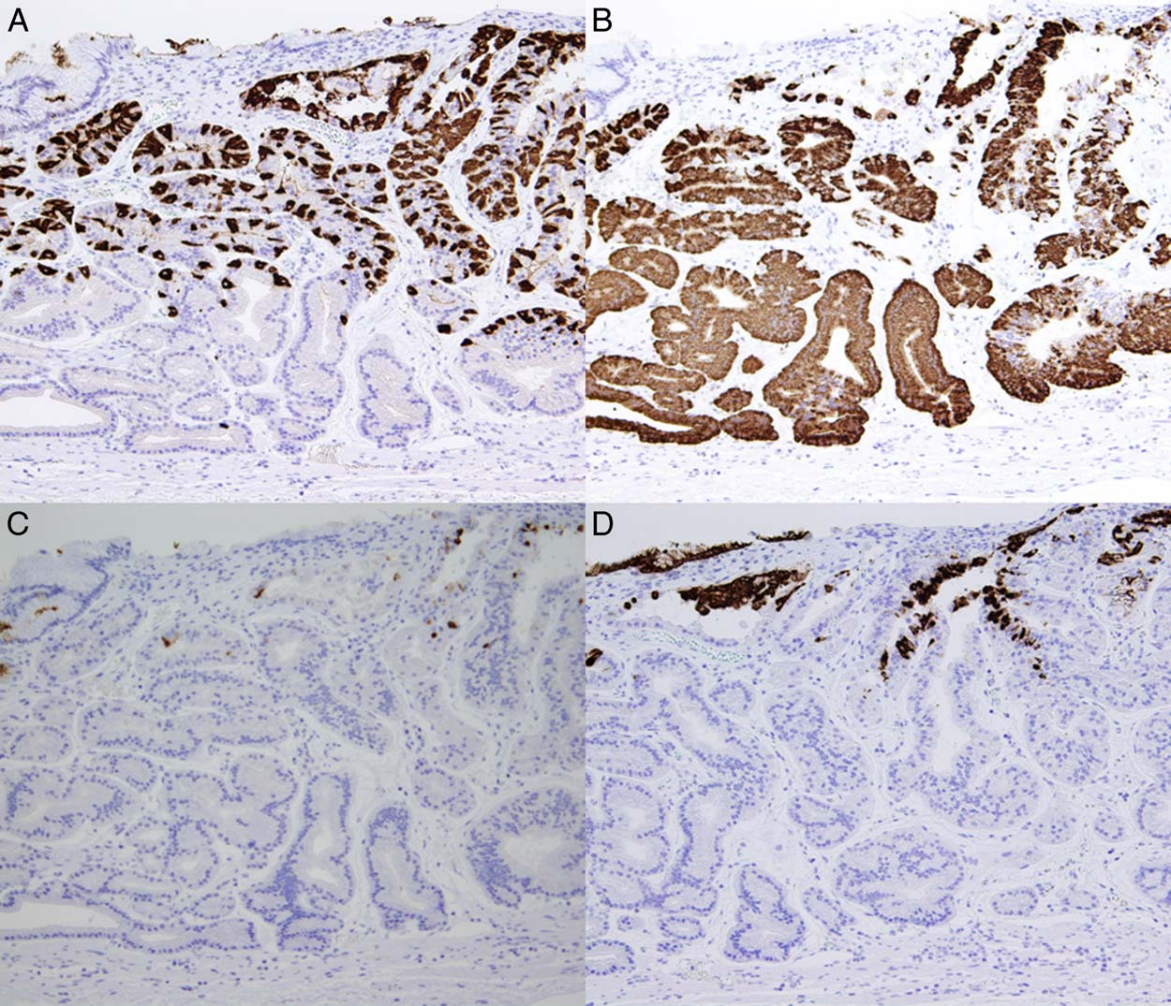
Immunohistochemistry specimen for gastric carcinoma of the fundic gland type. (A) Pathological examination of the specimen showed that pepsinogen 1 was localized in the mucosa. (B) Pathological examination of the resection specimen showed that MUC6 was confined to the mucosa. (C) Tumor cells were positive for H+/K+-ATPase immunostaining. (D) Tumor cells were negative for MUC5AC.

Thirty-two years after the eradication of *HP*, a 5 mm white-tone elevated lesion was found on the anterior wall of the upper part of the gastric body with dendritic vessels within the elevated area (Fig. 1A). Narrow-band imaging and weak magnification revealed surface microstructure, open gland orifices and interorbital spaces, and dendritic vessels (Fig. 1B). Atrophy of the gastric mucosa was C-1 according to the Kimura–Takemoto classification, indicating minimum atrophy. A biopsy of the elevated area showed an irregularly shaped glandular duct below the atypical epithelium, and a diagnosis of GCFGT consisting of atypical cells similar to the main cells was made. A whole-body computed tomography scan showed no evidence of distant or lymph node metastasis. Therefore, ESD was performed for GCFGT (Fig. 1C). The lesion was 8 mm, fading, and Type 0-IIa. The superficial layer was covered by non-neoplastic mucosa, and the number of tumor cells increased similar to the main cells, mainly in the deeper layers of the mucosa, with irregular branching structures, adhesions, and cyst-like dilatations (Fig. 1D). Furthermore, the final pathological diagnosis was U, Type 0-IIa, 8 mm, GCFGT, pT1a, UL0, Ly0, V0, HM0, and VM0 (Fig. 1D). Immunohistochemical staining was positive for pepsinogen 1 (Fig. 2A), MUC6 (Fig. 2B), H+/K+-ATPase (Fig. 2C), and negative for MUC5AC (Fig. 2D). The post-ESD course was uneventful, as shown by esophagogastroduodenoscopy performed 2 months, 8 months, and 1 year after ESD.

## Discussion

In this case, *HP* was eradicated in 1990, and GCFGT developed 32 years later. To the best of our knowledge, this is the longest case in which gastric cancer appeared after *HP* eradication. *HP* eradication therapy for a duodenal ulcer was first reported in 1990^10^, supporting that this is the longest case.

Take *et al*. monitored 2737 patients after the successful eradication of *HP* for up to 21.4 years and found 68 cases of gastric cancer during the observation period. Ten years or more after the eradication of *HP*, the incidence of subsequent gastric cancer was higher in the severe atrophy group (0.67%/year) than in the mild atrophy group (0.15%/year)^11^. In the present case, a patient with a history of duodenal ulcer and mild atrophy developed GCFGT 32 years after the eradication of *HP*.

A non-neoplastic epithelium covering cancerous tissue has been previously reported as a specific histological feature of gastric cancer after successful *HP* eradication^12^. Moreover, 50 and 5% of gastric cancers that develop >10 and <10 years after *HP* eradication, respectively, are reportedly covered by a non-neoplastic epithelium^13^. Kamada *et al*.^5^ reported that the characteristics of gastric cancer after successful *HP* eradication therapy are lesions <20 mm in size, located in the lower middle part of the stomach, microscopically depressed, and differentiated in histological type. In contrast, Take *et al*.^11^ reported that diffuse gastric carcinoma is a characteristic feature of gastric carcinoma that developed more than 10 years after *HP* eradication. In this case, the boundary of GCFGT was unclear, and diagnosing the extent of the carcinoma was difficult. It is necessary to consider the presence of diffuse gastric cancer and GCFGT and recognize that the surface of the cancerous tissue is covered by a non-neoplastic epithelium and presents a gastritis-like appearance when gastric cancer is detected more than 10 years after *HP* eradication.

GCFGT was first reported by Tohda *et al*. in 2007^14^, and it presents on conventional esophagogastroduodenoscopy as a submucosal tumor-like raised lesion, with faded and white tone, dilated resinous vessels, and no atrophy of the background mucosa^15^. Differential diagnoses include gastric submucosal tumor, neuroendocrine tumor, and gastric fundic gland polyp.

Overall, 40% of GCFGT were *HP*-positive^16^. Therefore, the presence or absence of *HP* infection in the development of GCFGT remains unclear. The fact that this patient experienced GCFGT 32 years later indicates that GCFGT may not be directly related to *HP* infection.

Because this patient had a history of *HP* eradication, when esophagogastroduodenoscopy was performed for follow-up after *HP* eradication, regardless of the presence or absence of *HP* infection, it was necessary to examine the patient with a view to detect differentiated or diffuse-type early gastric cancer and GCFGT. In a report of GCFGT, acid secretion inhibitors were administered in seven of eight cases^15^; however, this present case has no history of long-term use of acid secretion inhibitors.

## Conclusion

We report a case of GCFGT occurring 32 years after *HP* eradication for duodenal ulcers. Therefore, the risk of gastric cancer should be considered even in cases deemed to be at low risk of developing gastric cancer after *HP* eradication.

## Ethical approval

This case was approved by the Ethics Committee of the International University of Health and Welfare Hospital (approval number: 23-B-21) and adhered to the principles outlined in the Declaration of Helsinki.

## Consent

Written informed consent was obtained from the patient for the publication of this case report and any accompanying images.

## Sources of funding

None.

## Author contribution

D.S.: wrote the manuscript; K.S., M.Y., H.O., T.O., Y.O., Y.N., H.O., Y.S., and Y.K.: reviewed the literature and contributed to manuscript drafting; Y.K.: was responsible for the revision of the manuscript for important intellectual content. All authors approved the final version of the manuscript to be submitted.

## Conflicts of interest disclosure

The authors declare no conflicts of interest.

## Research registration unique identifying number

This paper is case report. The authors do not need to register this work.

## Guarantor

Daisuke Suto and Kiichi Sato.

## Data availability statement

The datasets will be shared upon reasonable request.

## Provenance and peer review

Not commissioned, externally peer-reviewed.
